# A dataset of publication records for Nobel laureates

**DOI:** 10.1038/s41597-019-0033-6

**Published:** 2019-04-18

**Authors:** Jichao Li, Yian Yin, Santo Fortunato, Dashun Wang

**Affiliations:** 1 College of Systems Engineering, National University of Defense Technology, Changsha, China; 2 Northwestern Institute on Complex Systems, Northwestern University, Evanston, IL USA; 3 Kellogg School of Management, Northwestern University, Evanston, IL USA; 4 McCormick School of Engineering, Northwestern University, Evanston, IL USA; 5 School of Informatics, Computing, and Engineering, Indiana University, Bloomington, IN USA; 6 Indiana University Network Science Institute (IUNI), Indiana University, Bloomington, IN USA

**Keywords:** Interdisciplinary studies, Careers, Complex networks

## Abstract

A central question in the science of science concerns how to develop a quantitative understanding of the evolution and impact of individual careers. Over the course of history, a relatively small fraction of individuals have made disproportionate, profound, and lasting impacts on science and society. Despite a long-standing interest in the careers of scientific elites across diverse disciplines, it remains difficult to collect large-scale career histories that could serve as training sets for systematic empirical and theoretical studies. Here, by combining unstructured data collected from CVs, university websites, and Wikipedia, together with the publication and citation database from Microsoft Academic Graph (MAG), we reconstructed publication histories of nearly all Nobel prize winners from the past century, through both manual curation and algorithmic disambiguation procedures. Data validation shows that the collected dataset presents among the most comprehensive collection of publication records for Nobel laureates currently available. As our quantitative understanding of science deepens, this dataset is expected to have increasing value. It will not only allow us to quantitatively probe novel patterns of productivity, collaboration, and impact governing successful scientific careers, it may also help us unearth the fundamental principles underlying creativity and the genesis of scientific breakthroughs.

## Abstract (summary)

**Table:** 

Design Type(s)	data integration objective • source-based data analysis objective • metadata search and retrieval objective
Measurement Type(s)	publication
Technology Type(s)	digital curation
Factor Type(s)	Knowledge Field • temporal_instant
Sample Characteristic(s)

Machine-accessible metadata file describing the reported data (ISA-Tab format)

## Background & Summary

Recent advances in the science of science have revealed a series of highly reproducible patterns governing the fundamental dynamic of science^1–3^, ranging from citation impact of papers^4–9^, career dynamics^10–22^, to teams and collaborations^23–27^. Although scientific elites have attracted ensuing interest from a wide range of disciplines^17,19–22,27–38^, spanning across sociology, economics, psychology, and physics, most quantitative analyses have focused on the prize-winning work alone, exploring the link between age and creativity^19,20^, and allocation of credits and recognition^27,30,32^. On the other hand, the rich patterns unveiled in qualitative studies^17,29^, including Zuckerman’s canonical work, vividly illustrates that the careers of scientific elites encompass projects well beyond their prize-winning work. Career-level analyses of these scientific elites have remained elusive, largely due to the difficulty in obtaining large-scale, high-resolution individual career histories.

Many studies have been devoted to building open-access datasets on scientific productivity and careers of scientists. For example, Vuong *et al*.^39^ introduced an open database on scientific output of Vietnamese researchers in social sciences and humanities; and Morrison *et al*.^40^ developed a name disambiguation method for inventors and assignees on 8.47 million patents. While these open-accessed datasets are mostly about ordinary scientists, researchers have also been interested in Nobel laureates. For example, Jones *et al*.^19^ collected a biographical dataset of 525 Nobel Prize winners, and Fortunato *et al*.^30^ curated data on dates of birth, the year of Nobel prizes and year(s) of publication(s) of prize-winning work. Chan *et al*.^41^ collected a dataset consisting of 34,448 publications of 192 Nobel laureates between 1970 and 2000 based on the Scopus dataset. Li *et al*.^42^ collected a fraction of papers published by Nobel laureates during the period of 1901–2012 using the Web of Science data. These efforts are highly complementary to the dataset presented here, highlighting the wide interest in the quantitative study of Nobel laureates.

There have also been practical utilities for such datasets. For example, Clarivate Analytics has developed tools that use similar proprietary publication and citation databases to predict future Nobel laureates. While the focus of Clarivate Analytics’ work is on predicting future laureates, the goal of our paper is to collect a comprehensive dataset capturing careers of individual Nobel laureates over the past century, which could then serve as an empirical starting point for future quantitative studies.

Here we build an open-access dataset on the scientific careers of Nobel laureates^43^. Despite a plethora of data capturing the various contributions of scientific elites, such information is often located in unstructured, isolated sources. Here, by combining unstructured information collected from Nobel Prize official websites, laureates’ personal and university websites, Wikipedia entries, and publication and citation records from the MAG, we constructed a unique dataset of career histories for nearly all Nobel laureates in Physics, Chemistry, and Physiology or Medicine from 1900 to 2016 (545 out of 590, 92.4%). We validated this dataset using four different approaches to ensure the reliability of our results, including comparison with manually collected CVs, selected Google Scholar (GS) profiles, additional affiliation information, and random selection of 60 Nobel laureates (20 for each field) for manual verification. The total data collection and validation procedure took more than 1000 hours.

The curated data could serve as critical input that feeds into several promising research directions. (1) The data make available quantitative patterns of productivity, collaboration, and impact governing the careers of scientific elites, offering a unique opportunity to systematically identify quantitative signals tracing the careers of elite scientists. (2) Combining our datasets with publication records that capture the careers of ordinary scientists offers opportunities for an array of fascinating comparative studies. Such studies would deepen our understanding of the factors driving exceptional scientific careers, helping us answer the broad question of what makes great scientists great.

As a more concrete example, we present two new findings using the curated dataset in the associated commentary^44^. Briefly, we find that careers of Nobel laureates are characterized by remarkably similar patterns as those of ordinary scientists. For example, apart from the prize-winning paper, all other important works in Nobel careers closely follow the random impact rule^10,11^, a finding that is contrary to the common belief that Nobel laureates tend to do critical work early in their careers. Further, the laureates also show a tendency toward collaborative research in larger teams, which runs counter to the iconic image of lone geniuses making solo contributions.

These results only represent some initial examples of how such datasets can help advance our quantitative understanding of career dynamics. Indeed, our dataset may help uncover a set of reproducible principles underlying individual creativity, offering insights into the conditions and environments that best facilitate scientific creativity and the genesis of scientific breakthroughs.

## Methods

**Fig. 1:**
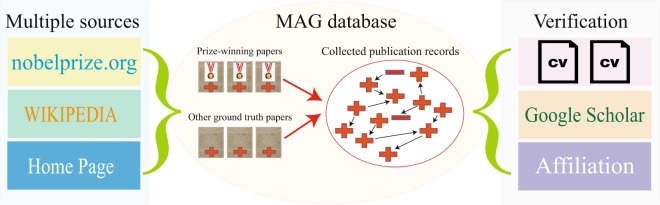
Data collection and validation framework. We first manually collected each laureate’s prize-winning paper and other “ground-truth” papers from multiple sources such as Nobel Prize official websites, Wikipedia, and their home pages, and matched these papers in MAG database as seeds. The entire publication record for each laureate is then collected through a “Seed + Expand” process. The red plus symbols indicate the laureate’s papers while the red minus symbols represent noise papers. The obtained dataset is validated with external sources, including CVs, GS profiles, affiliation information, and manual verifications.

We constructed the publication records for almost all Nobel laureates in physics, chemistry, and physiology or medicine from 1900 to 2016 (545 out of 590, 92.4%). We first collected information manually from Nobel Prize official websites, their university websites, and Wikipedia. We then matched it algorithmically with big data, tracing publication records from the MAG database. Figure 1 shows the data collection framework. Next, we describe how we collected and reconstructed the data we used in the project.

### MAG dataset

The MAG data contains scientific publication records, interlinked through the citation relationships between them, together with information about authors, institutions, publication venues (e.g. journals or conferences), and fields of study^45^. The dataset is updated on a weekly basis and contains 174,910,379 papers, 210,983,748 authors, 228,843 fields of study, 4,028 conferences, 47,963 journals, and 25,558 institutions as of June 2018.

### The biographical website for Nobel laureates

One advantage of studying Nobel laureates is that there is a large amount of information available and it is well-maintained by different organizations. Here we make use of three important sources: the Nobel Foundation’s website, Wikipedia webpages for the laureates, and university websites for the laureates.

1. The Nobel Foundation’s website. The Nobel Foundation’s website (nobelprize.org) offers a rich source of information about Nobel laureates. It contains numerous site-accessible documents, photos, audiotapes, videotapes, films, and articles for each laureate.
2. Wikipedia pages. Wikipedia is a multilingual, web-based, free encyclopedia based on a model of openly editable and viewable content (https://www.wikipedia.org/). All Nobel laureates have dedicated pages which offer detailed biographical information including biographies, scientific activities, selected publication records, awards, and honors, etc.
3. University websites for Nobel laureates. Nobel laureates’ personal homepages or lab websites offer an official account of their career records. These websites usually highlight several selected publications by the Nobel laureates, and sometimes even include an expanded list of all publications and official CVs. Although university websites are not guaranteed to be up-to-date, the information listed on these websites is, in general, highly accurate.

### Identifying prize-winning papers

The availability of information about Nobel laureates creates an unprecedented opportunity to identify laureates’ prize-winning work and to systematically collect their publication records throughout their careers. For each Nobel laureate, we collected data on the timing of prize-winning work, prize motivation, the title and year of the Nobel lecture, and the author and institution information on prize-winning work if it was available.

**Fig. 2:**
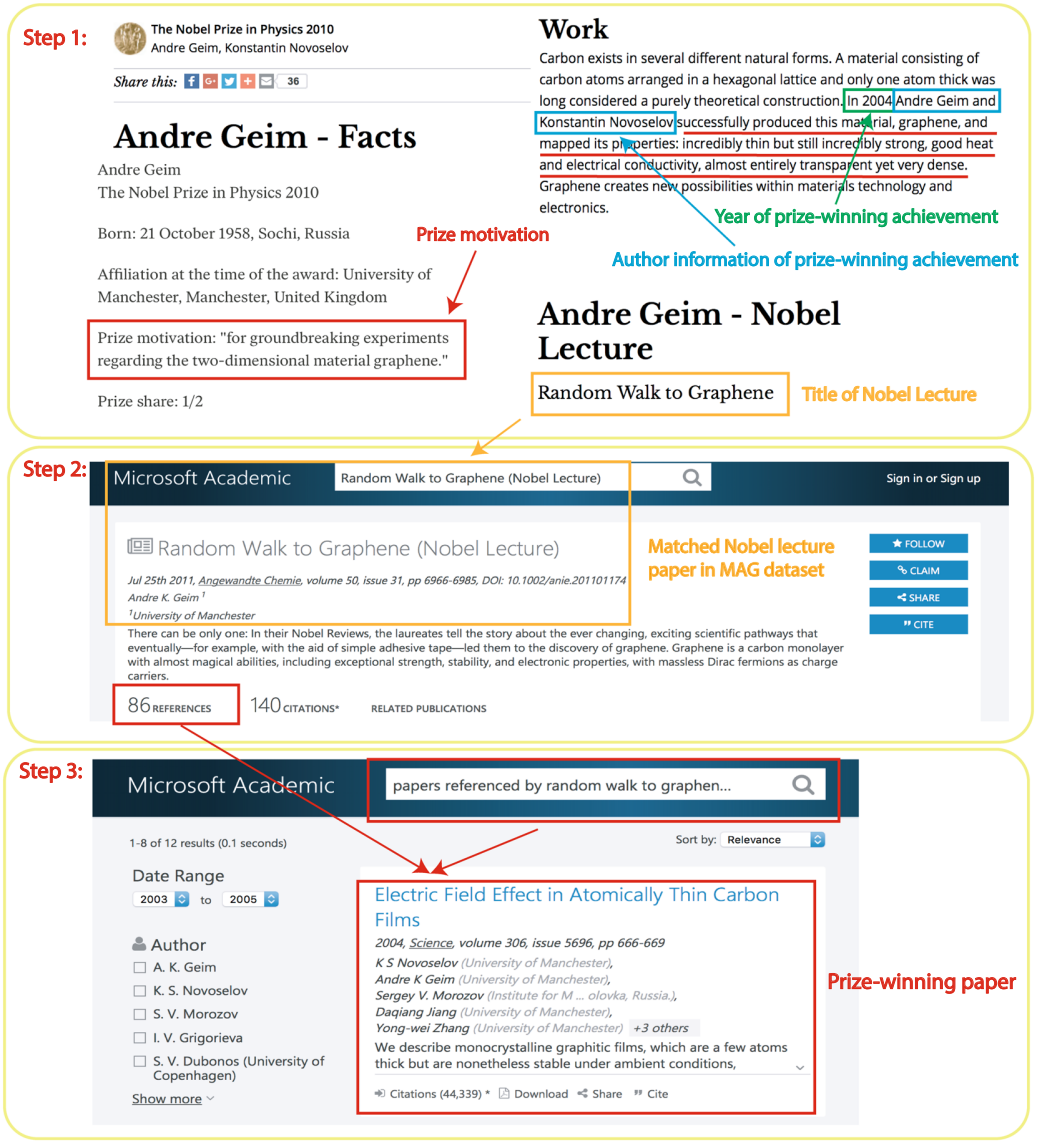
Procedure for identifying the prize-winning paper of 2010 Physics Nobel laureate Andre Geim. Step 1: The official website of the Nobel Prize, nobelprize.org, offers rich information for identifying the prize-winning works of Nobel laureates, including the year or period of the prize-winning achievement, the prize motivation, the title of the Nobel Lecture, etc. Step 2: We can obtain detailed information on the Nobel Lecture in the MAG dataset. Step 3: We can identify the prize-winning paper from the reference of the Nobel Lecture using information derived in Step 1.

The Nobel lecture provides detailed information about the prize-winning work. As such, the prize-winning papers are usually cited as references to the Nobel lecture. We collected all the references of Nobel lectures and manually collected the prize-winning papers identified in each Nobel lecture. For those that were not mentioned specifically in these lectures, we classify the reference of the Nobel lecture as a prize-winning paper if it satisfies all of the following criteria: (1) The Nobel laureate is in the author list of the reference paper, i.e., the paper has at least one author with the same last name and first name (or first initial if the full first name is missing) with the Nobel winner. For an author with a middle name or middle initial, such information must be consistent as well; (2) The paper was published within the same period of prize-winning papers; (3) Institution and co-author information of the reference is consistent with other auxiliary information about the prize-winning work; (4) The topic of the reference paper is consistent with the Nobel Prize motivation. If there are multiple papers that satisfy the criteria (1–4), we assume that prize-winning papers garner higher impact (measured by total citation). Following these procedures, we manually identified and collected the prize-winning papers for all laureates in our sample (Fig. 2).

In rare cases where prize-winning papers cannot be reliably identified from the references of the Nobel lectures, we consulted other sources: (1) Nobel laureates’ Wikipedia page usually offers a detailed personal biography, which contains their prize-winning contributions and mentions of prize-winning papers. (2) Important works are usually selected by the Nobel laureates and displayed on their personal homepage or lab website, which can help us to identify the prize-winning papers. (3) Existing studies have collected some of the prize-winning papers. For example, Shen and Barabasi^27^ collected the prize-winning papers for Nobel laureates in Physics (1995–2013), Chemistry (1998–2013), Medicine (2006–2013), and Economics (1995–2013). Our data collection also takes into account these existing efforts.

We matched all prize-winning papers with the MAG database. Together, we gathered 874 prize-winning papers for 545 Nobel laureates, including 283 prize-winning papers for 193 laureates in Physics, 259 prize-winning papers for 163 laureates in Chemistry, and 332 prize-winning papers for 189 laureates in Medicine.

### Name disambiguation procedure

A key challenge in analyzing scholarly databases is to identify the individual(s) who wrote a paper and, conversely, to identify all of the works that belong to a given individual^46–48^. This seemingly simple task represents a major unsolved problem for information and computer sciences, and a major roadblock to studies of individual careers. Here we exploit one important feature of the MAG data. Indeed, one major advantage of the MAG dataset is that author profiles have been processed through a well-designed disambiguation algorithm that optimizes the accuracy of a profile^45,48,49^. That is, each disambiguated profile in MAG may not contain all the papers published by an individual, but papers that are included in the profile do belong to the same person with high accuracy. In other words, MAG may split one scientist’s publication record into several different profiles, optimizing accuracy at the expense of recall. Note that authors themselves can also claim and assemble different profiles into one (https://www.microsoft.com/en-us/research/project/academic/articles/microsoft-academic-uses-knowledge-address-problem-conflation-disambiguation/), offering additional crowd intelligence to assist in the disambiguation procedure. This also suggests that if we could intelligently combine these profiles, we may be able to curate individual profiles with both high precision and recall.

**Fig. 3:**
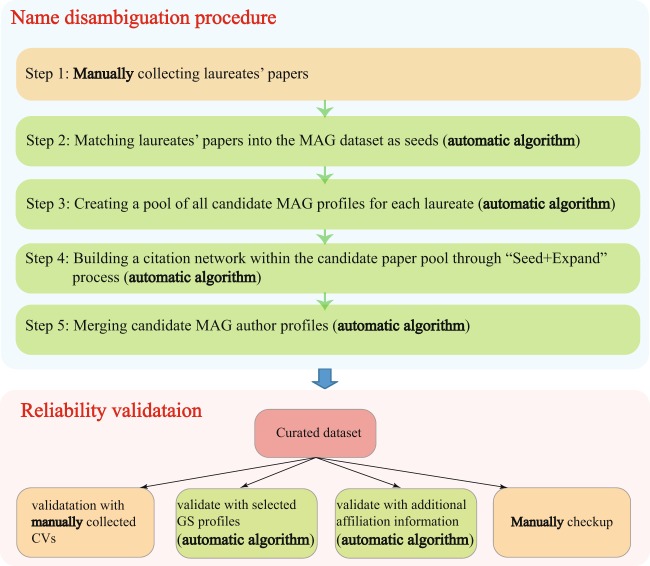
Name disambiguation procedure and reliability validation. We curated the Nobel laureates’ publication records through a name disambiguation procedure. The procedure consists of five steps: first, we manually collect laureates’ papers; next, the collected laureates’ papers are matched into the MAG dataset as seeds; we then create a pool of all candidate MAG profiles for each laureate through certain name matching rules; we build a citation network within the candidate paper pool through the “Seed + Expand” process; finally, all the candidate MAG author profiles are merged to obtain the curated publication record for each laureate. The reliability of the curated dataset is verified through four different approaches, including comparison with manually collected CVs, selected Google Scholar (GS) profiles, additional affiliation information, and random selection of 60 Nobel laureates (20 for each field) for manual verification.

**Fig. 4:**
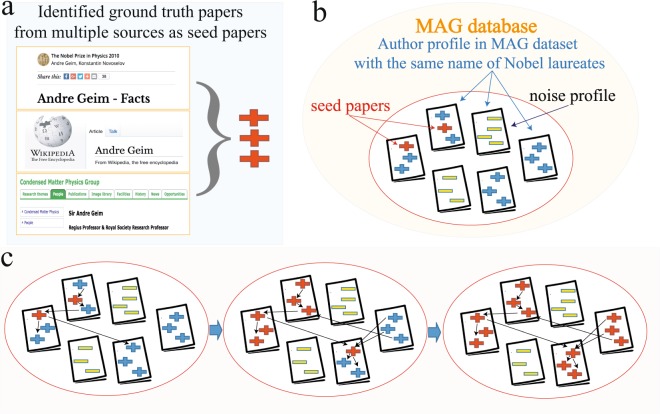
The name disambiguation process for Nobel laureates. (**a**) We first collected as many ground-truth papers as possible from multiple sources, including the Nobel Foundation’s website, Wikipedia’s website for Nobel laureates, and the laureate’s homepage. Then the ground-truth papers are matched into the MAG dataset as seed papers (red plus symbols). (**b**) A pool of all candidate MAG author’s profiles for each laureate are collected via name matching. Plus symbols indicate the laureate’s papers, and the red pluses denote the collected seed papers while the blue ones represent other potential papers by the laureate. The yellow minus symbol represents noise papers. (**c**) Name disambiguation process of the “Seed + Expand” algorithm. Starting with the original seed papers, we take candidate papers that have cited at least one of the seed papers as new seeds iteratively. The process stops when there is no paper in the pool to be added. We then merge all the candidate MAG author’s profiles having at least one paper in the ego citation network.

In this paper, we adopt a “Seed + Expand” procedure^50^, a method similar to label propagation in machine learning^51^ to merge various MAG author profiles into one. Figure 3 shows the detailed steps of the name disambiguation procedure, introduced as follows:

1. Manually collecting laureates’ papers. As part of identifying prize-winning papers, we have collected and identified several of the laureates’ papers, including the prize-winning papers, Nobel lecture papers, and other papers collected from their Wikipedia page, the Nobel official website, and their own homepage. These manually collected laureates’ papers are referred to as ground-truth papers.
2. Matching laureates’ papers into the MAG dataset as seeds. We then match these collected laureates’ papers into the MAG dataset using the following rules: Two papers are identical if they share the same Digital Object Identifier (DOI). In cases where the DOI is missing, they are considered to be the same paper if the following matching rules are satisfied: (1) the two papers were published within ±1 years; (2) the two papers have the same number of authors; (3) author sequence of the two papers is the same; (4) the text similarity, defined as the cosine similarity between the titles of the paper after removing stop words and punctuation, is higher than 0.75.
3. Creating a pool of all candidate MAG profiles for each laureate. We then created a pool of all candidate MAG profiles for each laureate according to the same rules followed by the literature^11^: (1) the author’s last name is identical to the laureate’s last name; (2) initial of the first name is identical to the laureate’s first initial. If an author’s full first name is available, it must be the same as the laureate’s; (3) for those authors who have middle names, the middle initial must be the same as the laureate’s. If the author’s full middle name is available, it also must be consistent with the laureate’s middle name. (4) For each laureate, all the publication records under the potential MAG author’s profiles constitute the candidate pool of papers for the laureate.
4. Building a citation network within the candidate paper pool through the “Seed + Expand” process. One assumption for the “Seed + Expand” process is that scientists are more likely to cite their own published work due to topical relevance or intellectual similarity between the papers. Thus, their own papers are usually connected through a citation network. Starting with the seed papers, we take candidate papers that have cited at least one of the seed papers and expand the pool of seeds iteratively. The process stops when there are no papers left in the candidate paper pool to be added to the network (Fig. 4).
5. Merging candidate MAG author profiles. For each Nobel laureate, we have a pool of candidate MAG authors. We then merge all the candidate MAG author profiles that have at least one paper in the citation network. Combining the merged MAG author’s profiles yields the entire publication history for each laureate.

Together, we curated publication records for each Nobel laureate, totaling 93,394 journal papers for 545 Nobel laureates.

### Reliability validation of the manually collected ground-truth papers

The manually collected ground-truth papers play an important role in the name disambiguation procedure. These ground-truth papers are matched into the MAG dataset as seeds, which are the starting point of the “Seed + Expand” process. Here we conducted cross-validation with additional information such as co-authors, affiliations, timing, a paper’s keywords, and even e-mail information, if available, to double check each manually collected laureates’ paper. We did a lot of tinkering to ensure the accuracy and correctness of the collected papers, trying to minimize the human errors accounted for in the “Seed + Expand” procedure. Nevertheless, although all these steps help reduce any potential human errors in the data curation process, thereby ensuring the accuracy of the collected data, readers should take note of the possibility that there may still be errors that remain unaccounted for given the manual processes.

## Data Records

### Data structure

We built an open-access dataset on publication records for Nobel laureates in Physics, Chemistry, and Medicine, which is available at Harvard Dataverse^43^. It contains four comma-separated values (CSV) files named “Prize-winning paper record,” “Physics publication record,”

“Chemistry publication record,” and “Medicine publication record.” The details are further described in Table 1.

**Table 1:** Dataset dimensions.

File	Lines	Short description
Prize-winning paper record	873	CSV format file containing prize-winning paper items for 545 Nobel laureates
Physics publication record	21504	CSV format file containing publication items for 193 Physics Nobel laureates
Chemistry publication record	42657	CSV format file containing publication items for 163 Chemistry Nobel laureates
Medicine publication record	29233	CSV format file containing publication items for 189 Medicine Nobel laureates

“Prize-winning paper record” contains information about prize-winning papers, including “Field,” “Laureate ID,” “Laureate name,” “Prize year,” “Title,” “Pub year,” “Paper ID,” and “Additional information” (Table 2). “Field” refers to the Nobel laureate’s field, i.e., Physics, Chemistry, or Medicine. We assign a unique “Laureate ID” for each laureate, and his/her name and prize-winning year are referred to as “Laureate name” and “Prize year.” “Title” and “Pub year” refers to the paper title and publication year of the prize-winning paper. “Paper ID” refers to the unique paper ID of each prize-winning paper in the MAG dataset. “Additional information” captures additional information about the prize-winning paper that may be relevant for interested readers. For example, John Macleod won the Nobel Prize in Physiology or Medicine in 1923 jointly with Frederick G. Banting for the discovery of insulin (https://www.nobelprize.org/prizes/medicine/1923/macleod/facts/); the experiment that resulted in the discovery was mainly conducted by Frederick Banting and Charles Best in John MacLeod’s laboratory in 1921 and was first published in the February 1922 issue of *The Journal of Laboratory and Clinical Medicine* under the names Frederick Banting and Charles Best; Macleod declined co-authorship because he considered it Banting’s and Best’s work (https://en.wikipedia.org/wiki/John_Macleod_(physiologist)).

**Table 2:** The data type for the prize-winning paper records.

Index	Format	Short description
Field	String	Nobel field for each laureate, i.e., Physics, Chemistry, or Medicine
Laureate ID	Integer	Unique ID for each Laureate
Laureate name	String	Name of the Nobel Laureate
Prize year	Integer	Year when the Laureate won Nobel Prize
Title	String	Title of the prize-winning paper
Pub year	Integer	Publication year of the prize-winning paper
Paper ID	Integer	Unique ID for each prize-winning paper
Additional information	String	Additional information refers to the prize-winning paper

The three files named “Physics publication record,” “Chemistry publication record,” and “Medicine publication record” contain the publication records of Nobel laureates for Physics, Chemistry, and Medicine, respectively. Each file includes the following data fields (Table 3): “Laureate ID,” “Laureate name,” “Prize year,” “Title,” “Pub year,” “Paper ID,” “DOI,” “Journal,” “Affiliation,” and “Is prize-winning paper.” Each data field is self-explanatory by its name, and fields with the same name as other tables follow the same data format and can be linked across tables. “DOI” and “Journal” refer to the DOI and the published journal for each collected paper. “Affiliation” refers to the Nobel laureate’s affiliation while publishing the paper. “Is prize-winning paper” shows whether the paper is prize-winning or not. If the paper is a prize-winning paper, the value of the item “Is prize-winning paper” is set as “YES,” otherwise it is set as “NO.”

**Table 3:** The data type for publication records of Nobel laureates for Physics, Chemistry, and Medicine.

Index	Format	Short description
Laureate ID	Integer	Unique ID for each Laureate
Laureate name	String	Name of the Nobel Laureate
Prize year	Integer	Year when the Laureate won Nobel Prize
Title	String	Title of the paper
Pub year	Integer	Publication year of the paper
Paper ID	Integer	Unique MAG ID for each paper
DOI	String	Digital Object Identifier (DOI) of the paper
Journal	String	Published journal of the paper
Affiliation	String	Nobel Laureate’s affiliation when publishing the paper
Is prize-winning paper	String	Whether the paper is a prize-winning paper or not

### Descriptive statistics

Table 4 shows the descriptive statistics resulting from the datasets in terms of different disciplines. We find that laureates’ productivity varies across different disciplines. The Chemistry laureates are the most productive, with each person publishing an average of 262 papers over their entire career, which is more than twice that of the Physics laureates’ average. We also find the mean age at which Nobel laureates did their prize-winning work is around 40, with no major age differences across disciplines. However, recognition for the prize-winning work always takes a long time, with Nobel laureates waiting an average of 17 years to win the Prize after making their prize-winning work.

**Table 4:** The descriptive statistics resulted from the datasets.

Statistics	Disciplines		
	Physics	Chemistry	Medicine
Collected laureates number	193	163	189
Average publications number	111	262	155
Average prize-winning paper number	1.5	1.6	1.8
Average age when making prize-winning papers	37.9	41.0	41.6
Average age when winning the Nobel Prize	55.6	58.4	57.9
Average recognition time after making the prize-winning work	17.7	17.4	16.3

## Technical Validation

### Reliability validation

To understand the reliability of the curated dataset, we take the following four different approaches to validate the data.

#### Validation with CVs of the laureates

We manually collected 30 laureates’ CVs from their personal websites, lab homepages, or university homepages which contain their full list of publications. Compared against these publication histories as the gold standard, our data shows a high precision and recall at 82.3% and 92.2% respectively.

#### Validation with GS profiles

Google provides scholar profiles for scientists to create, maintain and update their own publication records. Armed with Google’s proprietary name disambiguation algorithms, Google Scholar profiles may represent a comprehensive collection of individual publication histories. Keep in mind, however, that GS profiles tend to cover currently active scientists, and only a modest fraction of Nobel laureates have their own GS profiles. We collected GS publication records for 29 Nobel laureates. Since GS indexes not only journal publications but also conferences, patents, reports, meeting abstracts, talks, reviews, and even slides, here we consider only journal publications. Compared against the 29 GS profiles, our data shows precision and recall of 87.2% and 84.2% respectively.

#### Validation with additional affiliation information

More than 80% of publications we collected contain author affiliations, allowing us to use additional affiliation information to verify the reliability of our data. For each Nobel laureate, we first collected his/her affiliations from his/her Wikipedia and University homepage. Then we went through the curated publication list to check if the affiliation information recorded in the paper was consistent with the laureate’s career history. We find our data has high accuracy (97.6%).

#### Manual checkup

The MAG matched the biography information for a selected fraction of Nobel laureates from the Wikipedia to the author profiles. We can access this information from the MAG API (https://academic.microsoft.com/). We randomly selected 60 Nobel laureates (20 for each field) to manually check the reliability of our data.

**Fig. 5:**
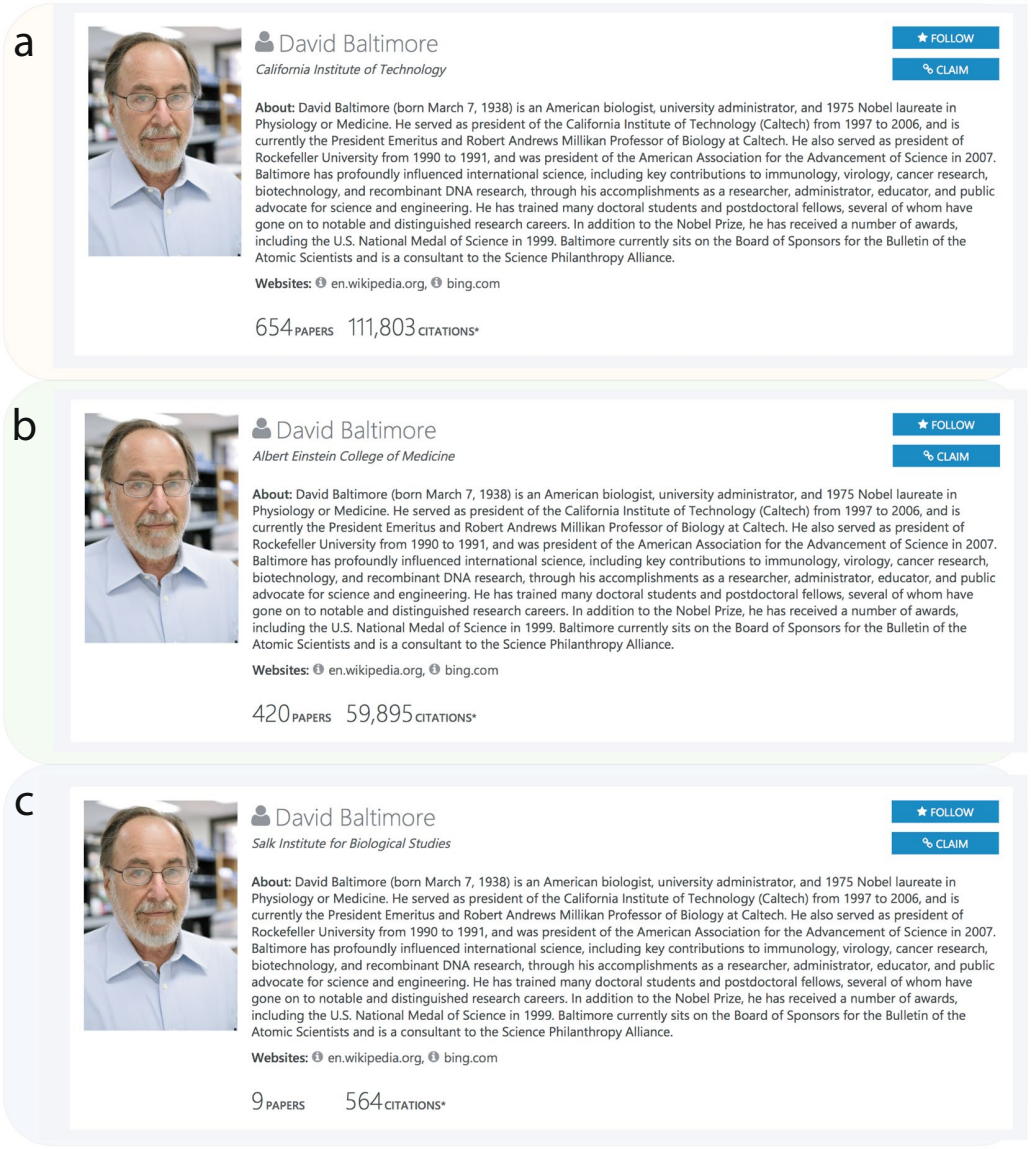
The three different MAG author profiles for Nobel laureate David Baltimore. MAG identified these as three different authors partly due to the difference in affiliations: California Institute of Technology, Albert Einstein College of Medicine, and Salk Institute for Biological Studies. We find that David Baltimore has indeed worked at all three places, suggesting the correct way of identifying all his papers is to merge these MAG profiles into one. [Image of David Baltimore was taken by Bob Paz and available for public use (https://en.wikipedia.org/wiki/David_Baltimore#/media/File:Dr._David_Baltimore2.jpg), and the image is licensed under the Creative Commons Attribution-Share Alike 3.0 license].

In Fig. 5, we illustrate this process through the example of David Baltimore, an American biologist who won the Nobel Prize in Physiology or Medicine in 1975. We identified and merged 24 MAG author profiles with the same name (David Baltimore), in which only three MAG author profiles contain more than five papers, and the rest contain only one or two papers each. Figure 5 shows the three main author profile pages of David Baltimore in the MAG API, suggesting that the three author profiles were considered different partly due to different affiliations: California Institute of Technology, Albert Einstein College of Medicine, and Salk Institute for Biological Studies. We further checked his affiliations, finding that David Baltimore worked at Albert Einstein College of Medicine, then moved to Salk Institute for Biological Studies in La Jolla as an independent research associate, and he also served as the president of the California Institute of Technology (Caltech). We manually checked all the papers under the 21 different MAG author profiles and calculate the accuracy. Manually counting through the papers, we find the accuracy of our data is 97.3% within the 60 careers we selected.

### Validation of the reproducibility of the dataset and method

In this paper, we adopt a “Seed + Expand” procedure to solve the name disambiguation problems of Nobel laureates. The proposed method is a hybrid procedure consisting of five steps, combining manual process and automatic algorithm. It is worth noting that only step one needs a manual collection of laureates’ papers, and the other four steps (from step two to step five) can be fulfilled automatically with established procedures and rules. The highly automated process of our method guarantees the reproducibility of the dataset and method.

Together, we present a novel systematic dataset recording career information of Nobel laureates in science. Although the curated data offers, to our knowledge, one of the most comprehensive publication records of Nobel laureates so far, the goal of publishing this dataset is to allow all interested readers to help further refine and improve the quality of the data.

## ISA-Tab metadata file

Download metadata file
